# Do initial concentration and activated sludge seasonality affect pharmaceutical biotransformation rate constants?

**DOI:** 10.1007/s00253-021-11475-9

**Published:** 2021-08-23

**Authors:** Tamara J. H. M. van Bergen, Ana B. Rios-Miguel, Tom M. Nolte, Ad M. J. Ragas, Rosalie van Zelm, Martien Graumans, Paul T. J. Scheepers, Mike S. M. Jetten, A. Jan Hendriks, Cornelia U. Welte

**Affiliations:** 1 Department of Environmental Science, Institute for Water and Wetland Research, Radboud University, Nijmegen, The Netherlands; 2 Department of Microbiology, Institute for Water and Wetland Research, Radboud University, Nijmegen, The Netherlands; 3 Faculty of Science, Open Universiteit, Heerlen, The Netherlands; 4 Radboud Institute for Health Sciences, Radboud University Medical Center, Nijmegen, The Netherlands

**Keywords:** Kinetics, Sorption, Organic micropollutants, Wastewater treatment plants, Bacterial community, Nitrification

## Abstract

**Abstract:**

Pharmaceuticals find their way to the aquatic environment via wastewater treatment plants (WWTPs). Biotransformation plays an important role in mitigating environmental risks; however, a mechanistic understanding of involved processes is limited. The aim of this study was to evaluate potential relationships between first-order biotransformation rate constants (*k*_*b*_) of nine pharmaceuticals and initial concentration of the selected compounds, and sampling season of the used activated sludge inocula. Four-day bottle experiments were performed with activated sludge from WWTP Groesbeek (The Netherlands) of two different seasons, summer and winter, spiked with two environmentally relevant concentrations (3 and 30 nM) of pharmaceuticals. Concentrations of the compounds were measured by LC–MS/MS, microbial community composition was assessed by 16S rRNA gene amplicon sequencing, and *k*_*b*_ values were calculated. The biodegradable pharmaceuticals were acetaminophen, metformin, metoprolol, terbutaline, and phenazone (ranked from high to low biotransformation rates). Carbamazepine, diatrizoic acid, diclofenac, and fluoxetine were not converted. Summer and winter inocula did not show significant differences in microbial community composition, but resulted in a slightly different *k*_*b*_ for some pharmaceuticals. Likely microbial activity was responsible instead of community composition. In the same inoculum, different *k*_*b*_ values were measured, depending on initial concentration. In general, biodegradable compounds had a higher *k*_*b*_ when the initial concentration was higher. This demonstrates that Michealis-Menten kinetic theory has shortcomings for some pharmaceuticals at low, environmentally relevant concentrations and that the pharmaceutical concentration should be taken into account when measuring the *k*_*b*_ in order to reliably predict the fate of pharmaceuticals in the WWTP.

**Key points:**

• *Biotransformation and sorption of pharmaceuticals were assessed in activated sludge.*

• *Higher initial concentrations resulted in higher biotransformation rate constants for biodegradable pharmaceuticals.*

• *Summer and winter inocula produced slightly different biotransformation rate constants although microbial community composition did not significantly change.*

**Graphical abstract:**

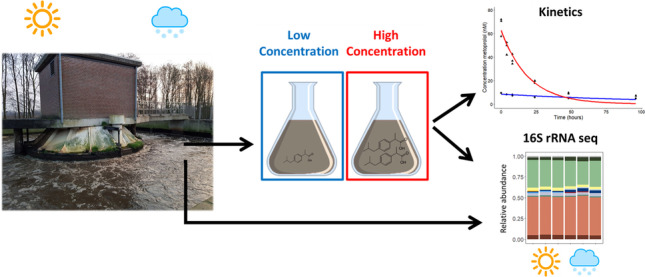

**Supplementary Information:**

The online version contains supplementary material available at 10.1007/s00253-021-11475-9.

## Introduction

Tonnes of pharmaceuticals are prescribed annually, which partly find their way to the aquatic environment via sewage systems and wastewater treatment plants (WWTPs) (Fent et al. 2006). The concentration of these pharmaceuticals in the aquatic environment is generally low (ranging from nM to µM concentrations), and thus, they are considered as organic micropollutants (OMPs). Even at such low concentrations, they can elicit large adverse effects on aquatic life including long-term or short-term toxicity (e.g. Fent et al. 2006; Santos et al. 2010). In WWTPs, the fate of a pharmaceutical is mostly influenced by microbial biotransformation (Verlicchi et al. 2012), a process which is still difficult to capture accurately with modelling approaches. Statistical quantitative structure activity relationship (QSAR) models can describe approximately 50% of the variability in biotransformation rate constants (e.g. Nolte et al. 2020). This explains for a large part why some OMPs, including pharmaceuticals, are better removed than others. However, the question remains why some WWTPs remove specific pharmaceuticals better than others. Statistical meta-analyses either find a low explanatory value for the removal efficiency of a large structurally diverse set of OMPs, including pharmaceuticals (17%; Douziech et al. 2018), or perform better but are limited to specific groups of OMPs (*R*^2^_*adj*_ ranged from 0.35 to 0.73; Wang et al. 2020). Due to the limited knowledge on general principles influencing microbial conversion of pharmaceuticals, biotransformation is yet not modelled mechanistically in fate models such as SimpleTreat, which is part of the European Union System for the Evaluation of Substances (Franco et al. 2013; Struijs 2014).

Instead, biotransformation rate constants used in fate models are typically estimated with standard methods such as OECD ﻿tests [OECD (1981) TG 302 series, OECD (1992) TG 301 series, OECD (2006) TG 310, OECD (1981) TG 302 series, OECD (2008) TG 314B and OECD (2001) TG 303A, in case of SimpleTreat]. It has to be noted that these tests are not designed for this purpose, but rate constants can nevertheless be derived from percentage removal or biodegradability categories (Struijs 2014). When OECD test outcomes are used to predict the fate in WWTPs, the environmental realism of the biotransformation rate constants is questionable as the test outcomes apply to specific incubation conditions with a long time duration and the microbial community composition of the inoculum varies (Goss et al. 2020; Kowalczyk et al. 2015; Li and McLachlan 2019; Rücker and Kümmerer 2012). In most OECD screening tests, pharmaceutical concentrations of 10–400 mg of carbon per liter may be applied (Kowalczyk et al. 2015), which is far above environmentally relevant concentrations (in the range of µg L^−1^) and this may affect biotransformation rate constants. Recent studies showed that first-order biotransformation rate constants depend on initial concentration in activated sludge treatment plants (Nolte et al. 2020) and in biofilm reactors (Svendsen et al. 2020). Biotransformation rate constants are often calculated as pseudo-first order constants (hereafter referred to as *k*_*b*_, Schwarzenbach et al. 2005), assuming no saturation of enzymes occurs and the maximum velocity of the reaction is not reached (for more information, see “Theory”). In the calculation of *k*_*b*_, an effect of concentration is already taken into account as the decrease in concentration over time depends on a rate constant and the concentration. Svendsen et al. (2020) showed that for some pharmaceuticals, *k*_*b*_ did not follow typical Michaelis–Menten kinetics at low, environmentally relevant concentrations (1–10 µg L^−1^) as *k*_*b*_ is expected to stay constant and the removal rate decreases over time as a result of the changing concentration. Instead, the *k*_*b*_ of some pharmaceuticals such as metoprolol increased with increasing initial concentration.

The concentration of pharmaceutical compounds in the influent of WWTPs can vary between seasons as a result of different consumption patterns by the population and a different amount of rainfall (Caucci et al. 2016; Di Marcantonio et al. 2020). Furthermore, WWTP operational parameters (i.e., solid retention time and hydraulic retention time) and other environmental conditions such as temperature have seasonal variability (Awolusi et al. 2018; Limpiyakorn et al. 2005). As a consequence of these changes, different microbial community compositions have been found in same WWTPs at different seasons. For example, ammonia oxidizing bacteria (AOB) were often found more abundant during summer than during winter and this was correlated to higher nitrification rates and lower ammonium concentration in the effluent of WWTPs (Awolusi et al. 2018; Ju et al. 2014; Liu et al. 2019; Wang et al. 2012). Previous experiments have observed a positive correlation between the *k*_*b*_ of specific pharmaceuticals and ammonium removal or nitrification rate (Fernandez-Fontaina et al. 2012; Helbling et al. 2012). Furthermore, higher abundance of specific microbial taxa has been correlated to a higher *k*_*b*_ and removal efficiency of some pharmaceuticals in AS (Helbling et al. 2015; Kim et al. 2017).

The aim of this study was to determine the influence of initial pharmaceutical concentration and sampling season of AS inocula on the *k*_*b*_ of pharmaceutical compounds. To our knowledge, this is the first time that the effect of pharmaceutical concentration on *k*_*b*_ is experimentally studied in activated sludge by means of a batch test. We selected nine pharmaceuticals based on high prescription rates in Europe (Fent et al. 2006) and/or with a high priority to monitor due to adverse environmental effects (i.e., pharmaceuticals on EU and Dutch watch lists): acetaminophen, metformin, diclofenac, metoprolol, terbutaline, diatrizoic acid, fluoxetine, and carbamazepine. Four-day batch assays were performed with AS inocula of the same WWTP taken at different seasons (summer and winter). Biotransformation rate constants and solid water partition coefficients (*k*_*b*_ and *k*_*d*_, respectively) were generated at two environmentally relevant concentrations (3 and 30 nM; 0.4–1.8 and 3.9–18.4 µg L^−1^). Summer and winter samples were analyzed on nitrification activity (including *amoA* gene abundance) and microbial community composition. We hypothesized that (i) a higher spiked concentration in the range of 3 to 30 nM result in a higher *k*_*b*_ and (ii) inocula taken at different seasons in the same WWTP have different characteristics (i.e., microbial community, nitrification activity, amoA abundance, and pharmaceutical concentration) that will affect the biotransformation rate constants of pharmaceuticals, with higher rate constants during summer.

## Materials and methods

### Chemical selection and experimental setup

We conducted biotransformation assays with nine pharmaceuticals (Table S1) twice: in June 2019 (summer) and December 2019 (winter). The log *K*_*ow*_ was obtained from Drugbank (Wishart et al. 2006), while information on the solid form of the pharmaceuticals was provided by the supplier (SI 1).

**Fig. 1:**
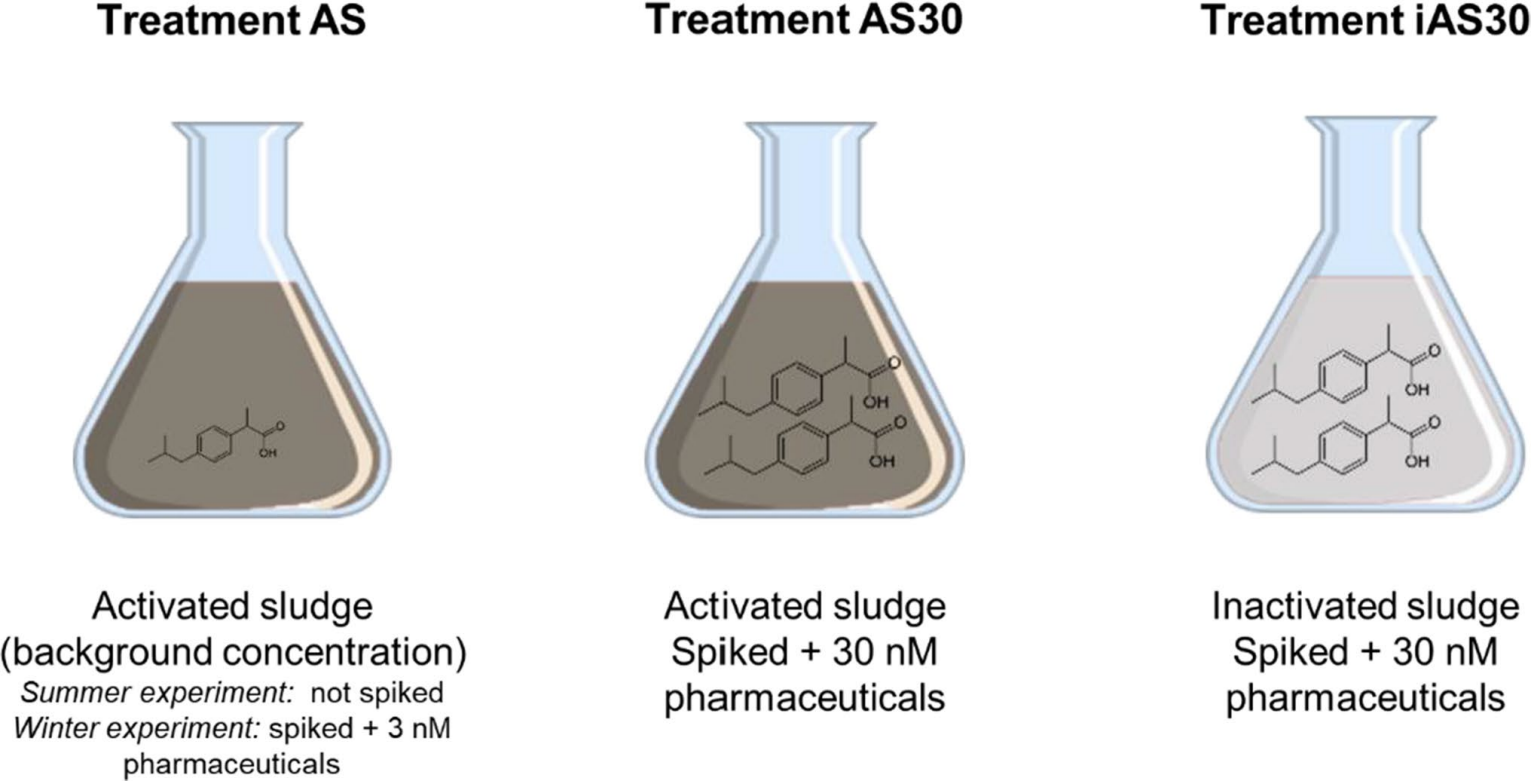
Treatments used in the summer and winter experiments: AS was either non-spiked, only including the background concentration of pharmaceuticals (summer experiment), or spiked with 3 nM pharmaceuticals (Winter experiment); AS30 is the AS treatment that was spiked with 30 nM of pharmaceuticals; and iAS30 is the inactivated sludge treatment that was spiked with 30 nM of pharmaceuticals

The removal of pharmaceuticals was tested for a duration of 96 h, which is sufficient to observe short term activity due to the high microbial activity of undiluted activated sludge samples (e.g. Helbling et al. 2010). As the AS samples were undiluted and microorganisms were not washed in order to obtain environmental realism of the test, no lag phase was anticipated on. We tested three treatments in summer and winter AS inocula (Fig. 1):

1. “AS” treatment: where we evaluated pharmaceutical biotransformation at low concentrations in AS. In the summer experiment, we measured the removal of background concentrations and in the winter experiment we spiked 3 nM of each pharmaceutical.
2. “AS30” treatment: where we evaluated pharmaceutical biotransformation a high concentration in AS. To do that, we spiked with 30 nM of each pharmaceutical in both summer and winter experiments.
3. “iAS30” treatment: where we assessed the abiotic removal of pharmaceuticals in AS (i.e., sorption). For that we doubled autoclaved AS and spiked 30 nM of each pharmaceutical.

The biotransformation experiments were carried out in 120-mL glass bottles. AS samples were taken from WWTP Groesbeek (Gelderland, The Netherlands), identified as a national hotspot of OMPs (Vissers et al. 2017). This WWTP has a maximum hydraulic capacity of 900 m^3^/h, and it treats mostly domestic water (estimated 90%). The WWTP contains an aeration tanks with a sludge age of 12 days. Five liters of activated sludge were sampled in winter and summer times. The samples were immediately transported to the laboratory and stored at 4 °C for < 72 h till the start of the experiment (*t* = 0). Individual stock solutions were prepared in methanol for each pharmaceutical at a concentration of 3 mM. Afterwards, a mixture of the nine pharmaceuticals was prepared in methanol too (final concentration of each pharmaceutical: 0.3 mM). To minimize the potential effects of the methanol solvent, the spike mixture of pharmaceuticals (to a final bottle concentration of 3 or 30 nM) was added to each bottle prior to addition of sampled AS. Methanol was allowed to evaporate in a fume hood with gentle air circulation for approximately 30 min and subsequently 90 mL of AS or iAS were added to the bottles. Additionally, 50 mM HEPES (4-(2-hydroxyethyl)-1-piperazineethanesulfonic acid) was added in order to prevent acidification and maintain a pH around 7. Prior incubations to this experiment showed a drop in pH below 6 when no buffer was added to the activated sludge (data not shown). Ruiz et al. (2003) showed that a pH below 6 can lead to inhibition of nitrification, which has possible consequences for biotransformation rates as they have been previously linked to nitrification activity (Helbling et al. 2012). The bottles were manually mixed to allow for complete dissolution of pharmaceuticals and the first samples were taken (*t* = 0) and centrifuged to separate the sludge from the aqueous phase. Hereafter, samples were taken after 4, 8, 24, 48, and 96 h. Bottles were closed with cotton plugs to allow aeration and prevent contamination from the environment. The bottles were incubated at 15 °C and shaken at 200 RPM to ensure continuous mixing and aeration, so that a constant dissolved oxygen concentration of approximately 1 mg L^−1^ was maintained. All biotransformation assays were run in triplicates. Bottles were incubated in the dark in order to reflect WWTP conditions and to exclude photodegradation or growth of photoautotrophs. Double autoclaved inactivated sludge bottles (treatment iAS30) were prepared before the start of the experiment (and spiking) by autoclaving bottles at 121 °C and 103 kPa for 20 min and repeating this procedure after 24 h as previously described (Helbling et al. 2010).

### Wastewater analyses of the batch incubation

Temperature and pH were monitored every 24 h with a Metrohm Applikon pH meter (Schiedam, the Netherlands). In the beginning and at the end of the experiment (*t* = 0 and 96 h), the dissolved organic and inorganic carbon concentrations (DOC and DIC, respectively) were measured, as well as total suspended solids. Water samples for DIC analyses were filtered with glass-fiber filters (Ø 0.45 µm), stored at 4 °C, and measured within 24 h after sampling using infrared gas analyses (IRGA, ABB Advance Optima, Frankfurt, Germany; as in van Bergen et al. 2020). Samples for dissolved organic carbon (DOC) were filtered (Ø 0.45 µm) and analyzed using a Shimadzu TOC-L CPH/CPN Analyser (Shimadzu, Kyoto, Japan). TSS concentrations were determined according to standard methods (Baird et al. 2017). Ammonium, nitrite, and nitrate assays were performed with technical duplicates in 96-well microtiter plates. Ammonium was measured at 420 nm on a 96-well fluorescence spectrophotometer after reaction with OPA reagent (0.54% (w/v) ortho-phthaldialdehyde, 0.05% (v/v) β-mercaptanol, and 10% (v/v) ethanol in 400 mM potassium phosphate buffer (pH 7.3)) as previously described (In’t Zandt et al. 2018). The reaction volumes were adjusted to the 96-well plate: 10 µL of sample and 200 µL of OPA reagent. Nitrite was measured colorimetrically at 540 nm using the Griess assay. Afterwards, an incubation with vanadium chloride at 60 °C reduced all nitrate to nitrite and the sample was measured again at 540 nm (García-Robledo et al. 2014).

### Pharmaceutical analyses

Samples (2 mL) for measuring pharmaceutical concentrations were taken from the inoculum at 0, 4, 8, 24, 48, and 96 h after the start of the incubation. Additionally, we also took samples from the activated sludge in order to measure background concentrations of pharmaceuticals before spiking. Samples were centrifuged and the supernatant was stored at − 20 °C until chemical analysis. A detailed LC–MS/MS protocol can be found in the supplementary material, including the solid phase extraction (SPE) and liquid–liquid extraction (LLE) protocol and recovery efficiencies (SI1). In summary, SPE was performed using Oasis HLB 3 cc SPE cartridges (Waters, Milford, MA, USA) to recover the pharmaceuticals present in the samples. Methanol was used to elute the pharmaceuticals, and after evaporation, they were dissolved in 0.1% v/v formic acid. To extract metformin, a LLE was performed (Yoshida and Akane 1999) by adding acetonitrile and sodium dodecyl sulphate. Calibration standards (*n* = 6, 0.5–50 nM) were freshly prepared and extracted in the same way as the samples. For the analysis of all the extracts, liquid chromatography tandem mass spectrometry analysis (LC–MS/MS) was used. TargetLynx LC–MS/MS data acquisition software (Waters, Milford, MA, USA) was used for the linear curve fitting. Quadratic curve fitting (*y* = *ax*^2^ + *bx* + *c*) was applied for the quantification of metformin because standard concentrations of extracted metformin did not fit to a linear model. Optimized LC–MS/MS parameters are provided in the supplementary data (Table S2, S3; Figure S1).

### Biotransformation rate constants

#### Theory

Biotransformation rate constants are often assumed to follow Michaelis–Menten kinetics (Schwarzenbach et al. 2005), where the reaction rate is a result of enzyme binding, product formation, and dissociation of the enzyme–substrate complex (Michaelis and Menten 1913), therefore, depending on substrate concentration (Eq. 1).

1 $$V=\frac{Vmax\times \left[s\right]}{\left[s\right]+Km}$$

where *V* is the reaction rate (mol L^−1^ h^−1^), *V*_*max*_ is the maximum reaction rate (mol L^−1^ h^−1^), [*S*] is the substrate concentration (mol L^−1^), and *Km* is the substrate concentration where *V* is half of *V*_*max*_ (mol L^−1^). As pharmaceutical concentrations in the wastewater are very low, it can be assumed that the substrate concentration is far below *K*_*m*_ and the reaction rate is linearly proportional to the substrate concentration. Hence, biotransformation rate constants can be calculated via pseudo-first order biotransformation kinetics (Schwarzenbach et al. 2005):

2 $${C}_{t}={C}_{0}\times {e}^{-kb\ t}$$

where *C*_*t*_ is the concentration at time *t* (nM), *C*_0_ is the concentration at time 0 (nM), and *k*_*b*_ is the biotransformation rate constant (h^−1^). Theoretically, the *k*_*b*_ (that includes the effect of substrate concentration at each time point) should be constant when the substrate concentration is below *K*_*m*_*.*

#### Biotransformation analyses

The *k*_*b*_ was calculated based on the average concentration of three technical replicates over six timepoints (*n* = 6). Exponential decay models were fitted to the concentration over time (Eq. 2), and quality of the model fit was evaluated by visual assessment and by referring to the goodness of fit parameters *R*^2^ and the *p*-value. The *k*_*b*_ was estimated using R (Version 4.0.2; R Core Team, 2020) and the packages drc, nlme, and aomisc (Pinheiro et al. 2017; Ritz et al. 2015). For some pharmaceuticals, only part of the curve was used for model fitting, and prior to analyses, one outlier was removed from the data (SI 2). Next to first-order biotransformation rate constants, zero-order and second-order models were fitted as well, but first-order models showed the best overall fit, so we chose to further work with first-order *k*_*b*_ values, also for comparability reasons. When we did not observe a significant decrease in concentration, we assumed no biotransformation occurred. Furthermore, first-order biotransformation rate constants were correlated with pharmaceutical concentration.

#### Sorption analyses

To check whether gradual abiotic removal processes (hydrolyses, sorption) occurred in the inactivated sludge treatment, similar first-order models were applied for that treatment. When we found a significant rate constant, we subtracted this from the *k*_*b*_ of the activated sludge treatment in order to obtain the biotransformation rate constants, thereby excluding chemical processes. Additionally, the solid water partition coefficient (*K*_*d*_) for each chemical based on the inactivated sludge treatment were calculated, assuming instantaneous sorption, according to Eq. 3 (as in Helbling et al. 2012):

3 $${K}_{d}=\frac{\frac{co,spike}{co,aq}-1}{TSS}$$

where *K*_*d*_ is the solids partitioning coefficient (*K*_*d*_, L g^−1^), where TSS is the total suspended solids concentration in the sample g ss L^−1^. The value for *C*_*aq*_ was directly measured in the aqueous phase for each sample from the glass bottles (*t* = 0; nM). Assuming instantaneous sorption, *K*_*d*_ can be related to the known spike concentration (*C*_0,*spike*_; nM) and *C*_0,*aq*_. All average values are given with their SD (± 1SD).

### Molecular analyses

#### Sampling and DNA isolation

Samples were taken from the summer and winter inocula before the experiments (in technical triplicates) and from the bottles at the end of the experiments. Sampling consisted of pipetting 2 mL of activated sludge suspension after thorough mixing. Afterwards, samples were centrifuged for 4 min at 20,000 × *g*: supernatants were transferred to another tube for chemical analysis and pellets were stored at − 20 °C until further analysis. DNA was extracted and purified using the DNeasy PowerSoil kit (QIAGEN Benelux) following the manufacturer’s instructions. DNA concentrations were determined using the Qubit dsDNA HS Assay kit and a Qubit fluorometer, both from Thermo Fischer Scientific (Waltham, MA USA).

#### Bacterial 16S rRNA gene sequencing and analysis

DNA samples were submitted to Macrogen (Seoul, South Korea) for amplicon sequencing of the bacterial 16S rRNA gene hypervariable V3 and V4 region. Sequencing was performed on an Illumina Miseq. The primers used for amplification were Bac341F (5′-CCTACGGGNGGCWGCAG-3′) and Bac785R (5′-GACTACHVGGGTATCTAATCC-3′) (Klindworth et al. 2013). Subsequent analysis of the sequencing output files was performed with R version 3.4.1 (R Core Team 2020). Pre-processing of the sequencing data was done using the DADA2 pipeline (Callahan et al. 2016). Taxonomic assignment of the reads was done up to the species level when possible using the Silva non-redundant database version 128 (Yilmaz et al. 2014). Count data were normalized to relative abundances. Data visualization and analysis were performed using phyloseq and ggplot packages (McMurdie and Holmes 2013; Wickham and Wickham 2007). Chao1, Simpson, and Shannon diversity indices were calculated using the estimate richness function of the phyloseq package. Permutational multivariate analyses of variance (PERMANOVA) was performed using the adonis function of the vegan package (Dixon 2003). The main drivers of differences in the microbial community composition between summer and winter inocula were identified using the top 15 eigenvalues from PERMANOVA. *T*-tests were performed on individual taxa to check if their changes were significantly different between sample groups.

#### Quantitative PCR

The relative cq numbers of *amoA* gene/16S rRNA gene copy numbers in inocula and samples from experiment bottles spiked with 30 nM or no pharmaceuticals were obtained using quantitative PCR. The above primers were used for 16S rRNA gene amplification. For bacterial *amoA* gene amplification, the following primers were used: (5′-GGGGTTTCTACTGGTGGT-3′) and (5′-CCCCTCKGSAAAGCCTTCTTC-3′) (Rotthauwe et al. 1997). A detailed qPCR protocol can be found in the supplementary material (SI3).

## Results

### WWTP and incubation conditions

Table 1 gives an overview of the characteristics of AS inocula used for the summer and winter experiments. WWTP conditions such as oxygen concentration, DOC, and TSS were largely similar in summer and winter at the time of sampling. The water temperature at the WWTP was higher during summer, as well as the DIC concentration, while the concentration of NH_4_^+^ was higher during winter. During both experiments, samples were incubated at 15 °C, with an oxygen concentration of approximately 1 mg L^−1^ and a constant pH of 7 to prevent ionization changes. During both experiments, TSS slightly increased (3.6–4.1 g ds L^−1^). In general, the background concentration of pharmaceuticals measured in the AS in summer and winter was similar (Table 2), with exception of the concentration of metformin, which was almost four times higher in winter (40 nM).

**Table 1:** Activated sludge conditions prior to the summer and winter experiment

	Summer	Winter
Ammonium (µM)	168 ± 14	597 ± 51
Nitrate (µM)	2 ± 0.6	0
Oxygen (mg L^−1^)	1.3	1.5
Temperature (°C)	18.3	13.6
pH	6.5	7
TSS (g ds L^−1^)	3.9 ± 0.03	3.2 ± 0.1
DOC (mg L^−1^)	45.2 ± 11.4	40 ± 7.9
DIC (mg L^−1^)	19.1 ± 0.8	59.7 ± 2.3

**Table 2:** Pharmaceutical background concentrations, *k*_*b*_ constants (h^−1^, in case of activated sludge treatments) of summer and winter experiments per treatment. AS is the activated sludge treatment without spiking (summer) or spiked with 3 nM pharmaceuticals (winter) and AS30 is the activated sludge treatment spiked with 30 nM pharmaceuticals. All data points and models used to estimate *k*_*b*_ can be found in Figure S4 and details on the statistical analyses can be found in Table S3

Pharmaceutical	CAS-number	Background concentration in activated sludge (nM) ± 1SD		Spiked concentration (nM) ^a^	*k*_*b*_ (h^−1^) ± 1SD	
		Summer experiment	Winter experiment		Summer experiment	Winter experiment
Acetaminophen	103–90-2	9.1 ± 5.0	7.5 ± 10.5	0 3 30	ns na 0.24 ± 0.09^b^	na 0.43 ± 0.04^**^ 1.07 ± 0.39^b^
Carbamazepine	298–46-4	7.2 ± 0.2	7.4 ± 0.4	0 3 30	ns na ns	na ns ns
Diatrizoic acid	117–96-4	< LOD^c^	< LOD^c^	0 3 30	< LOD^c^ na ns	na ns − 0.001 ± 0.0002**
Diclofenac	15,307–86-5	3.6 ± 1.4	2.0 ± 0.2	0 3 30	ns na ns	na ns ns
Fluoxetine	54,910–89-3	2.4 ± 0.8	< LOD	0 3 30	ns na ns	na ns ns
Metformin	657–24-9	12.6 ± 1.2	38.9 ± 12.0	0 3 30	0.02 ± 0.01* na 0.31 ± 0.10*	na 0.55 ± 0.17* 0.64 ± 0.15*
Metoprolol	37,350–58-6	9.5 ± 0.12	10.1 ± 0.5	0 3 30	0.01 ± 0.003* Na 0.05 ± 0.01**	na ns 0.02 ± 0.01*
Phenazone	60–80-0	< LOD^c^	0.02 ± 0.01	0 3 30	< LOD Na 0.002 ± 5E-5*	na ns ns
Terbutaline	23,031–25-6	< LOD^c^	1.8 ± 1.0	0 3 30	ns na 0.04 ± 0.01*	na ns 0.01 ± 0.001**

*ns* the slope of the concentration over time graph was not significant (Fig. S4), *na* this was not tested, ns the rate constant *k*_*b*_ by the fitted model (Eq. 1) was not significant (*p* > 0.05)

^*^p ≤ 0.05, **p ≤ 0.01, and ***p ≤ 0.001

^a^In summer, the spiked concentration was 0 nM, while at Winter, the spiked concentration was 3 nM

^b^Due to fast biodegradation, *k*_*b*_ was based on three or four timepoints (*t* = 0–24 h); therefore, results are insignificant although a trend was observed (more information: see Fig. S4, table S4)

^c^The limit of detection (LOD) of the pharmaceuticals in our method are reported in Table S1

### Nitrification activity

As previously mentioned, the ammonium concentration in the winter inoculum was higher than in summer. All ammonium was completely consumed in active bottles (AS and AS30) in both experiments during the first day (Figure S2). Nitrification activity in summer and winter experiments was quantified via nitrate production since ammonium was probably also consumed by heterotrophic bacteria for biomass production. Nitrite was always below the detection limit (2.5 µM) in active bottles. In the summer experiment, the production of 140 µM nitrate was observed in the first 2 days. In the winter experiment, an increase of 274 µM nitrate was measured mostly during the last 3 days of the experiment. Bottles with autoclaved biomass did not produce any nitrate (all data shown in Supplementary Figure S3). The nitrification rate was 17 µmoles nitrate day^−1^ g TSS^−1^ in the summer experiment and 28 µmoles nitrate day^−1^ g TSS^−1^ in the winter experiment.

### *Solid water partition coefficients (K*_*d*_*)*

Solid water partition coefficients were calculated based on instantaneous sorption. We found the highest *K*_*d*_ for fluoxetine (0.6 ± 0.1 L g^−1^), followed by carbamazepine (0.03 ± 0.06 L g^−1^), diclofenac (0.02 ± 0.02 L g^−1^) and metoprolol (0.01 ± 0.2 L g^−1^). Results of the autoclaved inactivated sludge treatment showed that most pharmaceutical concentrations did not decrease in this treatment. Therefore, gradual sorption over time was limited and no chemical processes such as hydrolysis were observed. Only a small but significant decrease of diclofenac was observed in the inactivated sludge treatment, likely due to gradual sorption (0.006 ± 0.002 h^−1^, *p* < 0.05; Table S4).

### Biotransformation rate constants

Table 2 shows background concentrations of pharmaceuticals at the start of the summer and winter experiment and the biotransformation rate constants. No lag phase was observed before biotransformation of pharmaceuticals occurred. Metformin had high biotransformation rate constants in both experiments, and in the winter experiment, the concentration of metformin was below the limit of detection (LOD) within 48 h in both activated sludge treatments (spiked with 3 and 30 nM; Fig. S4B). The *k*_*b*_ of metformin increased at higher concentrations of pharmaceuticals in both experiments. Acetaminophen was not transformed at the lowest concentration in the summer experiment (9.1 nM). At the high concentration treatment (39.1 nM) in summer and in both the low and high concentration treatments (10.5 nM and 37.5 nM, respectively) in winter, acetaminophen was transformed within 24 h. After 24 h, the concentration increased again only in some replicates, which may be caused by a limitation of the measuring method at low concentrations (for more information, see SI 2). At the start of the summer experiment (*t* = 0 h, < 20 min after addition of pharmaceuticals), the concentration of acetaminophen was much lower than expected based on the theoretically added concentration and even below LOD in the winter experiment (Figure S4), indicating a rapid turnover or uptake by the cells. Metoprolol and terbutaline were biotransformed in both experiments at lower rates than acetaminophen and metformin. In the summer experiment, the *k*_*b*_ of metoprolol increased at a higher concentration of pharmaceuticals, while at the winter experiment biotransformation was only observed in the high concentration (spiked with 30 nM). Terbutaline was only biotransformed when activated sludge was spiked with a high concentration of pharmaceuticals (30 nM). Phenazone was not biotransformednor adsorbed during the winter experiment. In the summer experiment, we observed a low biotransformation rate (0.002 ± 5E − 5 h^−1^) when activated sludge was spiked with 30 nM pharmaceuticals. No biotransformation of carbamazepine, diclofenac, and fluoxetine was observed. Diatrioic was not biotranformed either and the concentration even increased slightly when spiked with a high concentration (30 nM) in winter time (− 0.001 h^−1^).

### *Relationship of k*_*b*_* with concentration and sludge seasonality*

In general, pharmaceuticals with a higher intial concentration at the start of the experiment showed a higher *k*_*b*_ value (*n* = 5 pharmaceuticals; difference between pharmaceuticals; Figure S5). Of these compounds, metformin and metoprolol show an increase in *k*_*b*_ with concentration (difference within pharmaceuticals). Small effects of seasonality on *k*_*b*_ were observed. In winter time, the *k*_*b*_ of acetaminophen was higher than in summer at a similar concentration. In winter time, the *k*_*b*_ of metformin was higher on average than in summer time, although standard deviation error bars overlap, the 95% CI did not, indicating a significant difference. Metoprolol and terbutaline had only a slightly higher *k*_*b*_ in summer than in winter time (note that Figure S5 shows the log *y*-axe). Phenazone was slightly biotransformedin summer, but not in winter time. Metformin and metoprolol showed a different exponential decrease over time depending on initial concentration in the experiment as a result of spiking and season (Table 2; Figure S4).

### Microbial community analysis

**Fig. 2:**
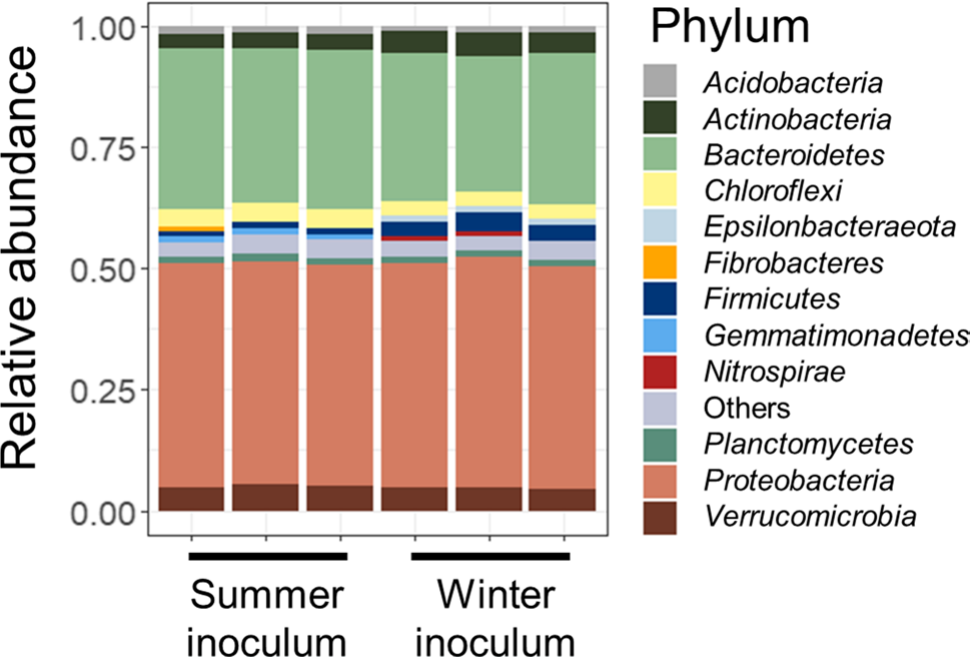
Phylum relative abundance of summer and winter inocula. No significant difference in microbial composition was observed between both inocula (PERMANOVA *p* > 0.05)

Microbial community analysis based on bacterial 16S rRNA gene sequencing was first performed on summer and winter inocula. Alpha diversity indices (Chao1, Simpson, Shannon) were not significantly different between summer and winter inocula (*p* > 0.05). PERMANOVA testing was used to calculate the significance of compositional differences between the groups. No significant difference was found between inocula (*p* > 0.05 at different taxonomic levels). The bacterial community composition of the two inocula is visualized in a relative abundance bar plot at the lowest taxonomic level (Fig. 2). Overall, the data show that the microbial communities of summer and winter inocula closely resemble each other. Zooming into the low differences between inocula (based on PERMANOVA calculated eigenvalues), the phyla contributing the most to variation were *Bacteroidetes* (higher in summer), *Firmicutes*, and *Actinobateria* (higher in winter). All of them were significantly different between both sampling times except for *Bacteroidetes*. At family level, the main contributors were *Burkholderiaceae* and *Rhodanobacteraceae* (higher in winter) and *Saprospiraceae*, *Cellvibrionaceae*, *Haliangiaceae* (higher in summer). Among the microorganisms previously related to acetaminophen degradation, the genera *Dokdonella*, *Flavobacterium*, *Acinetobacter*, and *Enterococcus* showed a significant (*p* < 0.05) relative abundance increase in winter correlating to a higher biotransformation rate constant of acetaminophen in our experiments (Figure S5; Figure S6). Microbial community analysis was also performed on biomass at the end of each experiment. The microbial community composition of the inocula and the biomass after 96 h of incubation were still very similar (PERMANOVA *p* > 0.05, data not shown). There were also no significant microbial community changes between experiment bottles with different pharmaceutical concentrations (PERMANOVA *p* > 0.05, data not shown). Differences in bacterial *amo*A gene abundance between inocula and at the end of the bottle incubations were quantified by qPCR and compared as relative cq values using the bacterial 16S rRNA gene cq as internal standard. A small but significant decrease (4%) in the relative cq number of *amo*A/16SrRNA was observed in the winter inoculum (Figure S7). This result aligns with the higher ammonium concentration found in Groesbeek WWTP due to decreased nitrification during winter. As a result of the higher ammonium concentration present at the beginning of the winter experiment, the nitrification rates in the bottles were higher than during the summer experiment.

## Discussion

In this study, we evaluated the effect of initial pharmaceutical concentration and AS seasonality on the biotransformation rate constants (*k*_*b*_) of nine highly prescribed pharmaceutical compounds. The *k*_*b*_ values of biodegradable pharmaceuticals were higher at the highest initial concentration, thereby confirming our hypothesis (i). More specifically, for pharmaceuticals metformin and metoprolol, a higher initial concentration increased their *k*_*b*_ (Table 2; Figure S5), even though in the calculation of *k*_*b*_ the concentration at each point in time is already taken into account (see “Theory”). For the other biodegradable compounds (acetaminophen in the summer experiment and terbutaline), the lowest concentration did not result in any biotransformation, which might indicate a mass transfer constrain as previously demonstrated with the well-known pesticide atrazine (Kundu et al. 2019). In general, biodegradable compounds with a higher background concentration in the AS had the highest *k*_*b*_ (Table 2). The *k*_*b*_ of acetaminophen and metformin were higher in the winter experiment. Nitrification rates were significantly higher in the winter experiment as a result of the higher ammonium concentration present at the beginning of the experiment (Figures S2; S3). However, this could not be clearly linked to a difference in *k*_*b*_ of acetaminophen and metformin. Metformin also had a three times higher concentration in winter, possibly explaining the higher *k*_*b*_. Acetaminophen degradation has been associated to heterotrophic bacteria rather than ammonia oxidizing organisms (De Gusseme et al. 2011). The relative abundance of putative acetaminophen degraders (*Flavobacterium*, *Dokdonella*, *Acinetobacter*, and *Enterococcus*) was significantly higher in the winter inoculum (Figure S6) (Akay and Tezel 2020; Chopra and Kumar 2020; Palma et al. 2018; Żur et al. 2018), likely explaining the increased *k*_*b*_ of acetaminophen in winter. The *k*_*b*_ of terbutaline, metoprolol, and phenazone were higher in summer than in winter, which could not be explained by concentration differences or microbial community composition. No significant differences in microbial community composition were observed between the summer and winter inocula (Fig. 2; PERMANOVA *p* > 0.05), opposed to our hypothesis (ii) and not between the start and the end of the experiment. In general, seasonal effects were not as straightforward as expected in our hypothesis (ii) and our results show pharmaceutical-dependent effects of sludge seasonality in biotransformation rate constants.

Biotransformation was observed as the predominant removal route for most pharmaceuticals in our experiments, except for fluoxetine, diclofenac, carbamazepine, and diatrizoic acid, which were slightly adsorbed or not removed at all. This lack of observed biotransformation was in line with previous studies, where these pharmaceuticals were either very slowly or not biotransformed or showed a high variability in biotransformation depending on the WWTP (Casas et al. 2015; Fernandez-Fontaina et al. 2012; Haiß and Kümmerer 2006; Kruglova et al. 2014; Petrie et al. 2015; Urase and Kikuta 2005). For diatrizoic acid, we even found slightly increasing concentrations in the winter experiment (Table 2; Fig. S4b). This phenomena has been previously explained by the reverse reaction of metabolites to the parent compounds (Gonzalez-Gil et al. 2019a, b; He et al. 2019; Tran et al. 2009) or to a desorption process (Gonzalez-Gil et al. 2018). Fluoxetine, diclofenac, carbamazepine, and metoprolol were instantaneously adsorbed to the activated sludge in accordance with other studies (Fernandez-Fontaina et al. 2016, 2012; Salgado et al. 2012) and corresponding with the highest *K*_*ow*_ values (Table S4). Gradual sorption only occurred for diclofenac. Acetaminophen and metformin showed high biotransformation rate constants, similar to other studies (e.g. Petrie et al. 2015; Poursat et al. 2019a), although no biotransformation was observed at the lowest spiked concentration for acetaminophen in summer. Metoprolol showed varying *k*_*b*_ values in other studies (Kasprzyk-Hordern et al. 2008, 2009; Svendsen et al. 2020) and terbutaline was biotransformed well in other studies (Bueno et al. 2012; Choubert et al. 2011). This corresponds to our findings, although no biotransformation was observed at the lowest spiked concentrations for metoprolol in winter and terbutaline in both seasons. Phenazone mostly shows a low *k*_*b*_ in other studies, similar to what we found (Casas et al. 2015; Onesios et al. 2009).

When the pharmaceutical concentration increased, *k*_*b*_ increased as well for metformin and metoprolol. A possible explanation for this is previous exposure of the microbial inoculum to the compound, which could lead to a higher biotransformation capacity (Poursat et al. 2019b). The biodegradable compounds metformin and metoprolol both occurred in a high background concentration in the activated sludge (Table 2). Similar effects of pharmaceutical concentration on *k*_*b*_ have been observed for metoprolol and citalopram in biofilms (Svendsen et al. 2020), with an increase in *k*_*b*_ at environmentally relevant concentrations. It is likely that Michealis-Menten theory is not always sufficient to describe biotransformation at low, environmentally relevant concentrations. Based on Michaelis–Menten kinetics, *k*_*b*_ should be constant at low concentrations, when the substrate concentration is far below *k*_*m*_ (substrate concentration at half the maximum reaction rate, also see “Theory”). We assume this is the case for environmentally relevant concentrations (in the nM range). A possible explanation for this is that Michealis-Menten theory is only developed for one substrate and one enzyme and the enzyme concentration is not considered to increase over time. However, in activated sludge, multiple enzymes could be responsible for the biotransformation of the same substrate and enzyme concentrations can increase due to microbial growth (Bilal et al. 2019, Khersonsky and Tawfik 2010). At higher concentrations (above environmentally relevant in the µM range), inhibition processes might start to play a role. For instance, Svendsen et al. (2020) found that the *k*_*b*_ of citalopram and metoprolol was decreasing at concentrations higher than environmentally relevant (25–300 µg L^−1^). Svendsen et al. (2020) furthermore found a decrease in *k*_*b*_ with increasing concentration for the ibuprofen, sotalol, atenolol, trimethoprim, and diclofenac in the concentration range 0.3–300 µg L^−1^. Wei et al. (2019) also found that the *k*_*b*_ of four pharmaceuticals (metronidazole, bezafibrate, ibuprofen, and sulfamethoxazole) was negatively influenced by concentrations ranging from 0.1 to 3 µM, which are notably higher than the concentrations tested in this study and likely caused inhibition of microbial processes, especially in case for antibiotics.

In a previous experiment, pharmaceutical biotransformation kinetics differed when inocula were taken from different WWTPs (Helbling et al. 2012). In our assays, we observed small *k*_*b*_ differences when the inocula came from the same WWTP at different seasons, summer and winter. The *k*_*b*_ of acetaminophen was higher in the winter experiment, when the relative abundance of putative acetaminophen degraders (*Flavobacterium*, *Dokdonella*, *Acinetobacter*, and *Enterococcus*) was significantly higher as well (Figure S6). The *k*_*b*_ of metformin was also higher in winter, likely due to a higher concentration of the pharamaceutical. A bacterium isolated from activated sludge (*Aminobacter* sp. affiliated with *Phyllobacteriaceae*) has been previously correlated with metformin degradation (Poursat et al. 2019a). However, the 16S rRNA gene of *Aminobacter* was absent from our amplicon dataset, indicating that metformin degradation is not limited to *Aminobacter* in activated sludge microbial communities. Other pharmaceuticals such as carbamazepine and diclofenac showed a higher *k*_*b*_ with increasing ammonium concentrations in previous 6-day batch experiments inoculated with nitrifying AS (Tran et al. 2009). Likewise, fluoxetine biotransformation has been previously linked to nitrifying activities in a bioreactor of HRT = 3.6 days (Fernandez-Fontaina et al. 2012). However, in our assays, these three pharmaceuticals did not biotransform at all. Terbutaline, metoprolol, and phenazone biotransformation rates were slightly higher during the summer experiment, where nitrification rates were lower. Interestingly, metoprolol biotransformation was inhibited by nitrification in a previous experiment with biomass from constructed wetlands (He et al. 2018). Overall, nitrification activity could not clearly be linked to increased biotransformation of pharmaceuticals in our experiment. Although there were no significant differences in total microbial community composition between both inocula (Fig. 2), there might have been differences in activity of microorganisms able to degrade these pharmaceuticals. Previous studies have reported different microbial composition between seasons (Ju et al. 2014; Liu et al. 2019), but other researchers including us did not observe that (Isazadeh et al. 2016). This shows that microbial seasonal changes might be dependent on each WWTP. In order to identify which exact microorganisms are responsible for all these pharmaceuticals biotransformation under WWTP conditions, further experiments are needed.

In conclusion, higher initial concentrations resulted in higher biotransformation rate constants for biodegradable pharmaceuticals. Specifically, we observed an effect of concentration (3–30 nM) on the biotransformation rate constants of the pharmaceuticals metformin and metoprolol. Therefore, Michaelis–Menten theory is not always sufficient to describe kinetics at very low, environmentally relevant concentrations, and consequently, the pharmaceutical concentration should be taken into account when predicting/measuring the *k*_*b*_ in WWTPs. In addition, we found differences in *k*_*b*_ of specific pharmaceuticals between the summer and winter experiment, but this could not be largely explained by microbial community composition, except for acetaminophen, whose higher *k*_*b*_ was explained by a higher relative abundance of putative acetaminophen degraders. Nitrification activity could also not clearly be linked to increased biotransformation of the other pharmaceuticals, so heterotrophic microbial activities might be responsible for that. Additionally, our test showed environmental realism, as the microbial community did not change during the experiment. Although the exact mechanisms influencing the concentration dependency of *k*_*b*_ are yet to be unraveled, this dependency can be used to model the *k*_*b*_ more accurately. Methods such as transcriptomics may help to unravel the effect of concentration on biotransformation, as it gives information on microbial activity.

## Supplementary Information

Below is the link to the electronic supplementary material.

Supplementary file1 (PDF 682 KB)

## Data Availability

All sequencing data were submitted to the GenBank database under the BioProject ID PRJNA641582 (https://www.ncbi.nlm.nih.gov/bioproject/641582). The data that support the findings of this study are available in Dans Easy (10.17026/dans-z4g-fe9h).
