# Supplementary material for: Do initial concentration and activated sludge seasonality affect pharmaceutical biotransformation rate constants?
Source: Appl Microbiol Biotechnol. 2021 Aug 23;105(16-17):6515–27. doi: 10.1007/s00253-021-11475-9 (PMC8403117; doi:10.1007/s00253-021-11475-9)
Supplement: Supplementary file 1 — Supplementary file1 (PDF 682 KB) [file 253_2021_11475_MOESM1_ESM.pdf]

Applied Microbiology and Biotechnology

Do initial concentration and activated sludge seasonality affect pharmaceutical biotransformation rate constants?

Tamara J.H.M. van Bergen[1]*, Ana B. Rios Miguel[2]*, Tom M. Nolte[1], Ad Ragas[1,3], Rosalie van Zelm[1], Martien Graumans[4], Paul Scheepers[4], Mike S.M. Jetten[2], A. Jan Hendriks[1], Cornelia U. Welte[2]

[1] Department of Environmental Science, Institute for Water and Wetland Research, Radboud University, Nijmegen, The Netherlands

[2] Department of Microbiology, Institute for Water and Wetland Research, Radboud University, Nijmegen, The Netherlands

[3] Faculty of Science, Open Universiteit, Heerlen, The Netherlands

[4] Radboud Institute for Health Sciences, Radboud University Medical Center, Nijmegen, The Netherland

* These authors contributed equally to the manuscript

# Address correspondence to Tamara van Bergen (t.vanbergen@science.ru.nl) and Cornelia Welte (c.welte@science.ru.nl); telephone: +31 24 3652952

SI 1. Extended methodology pharmaceuticals

Chemicals and reagents

Pharmaceutical standards were purchased in solid form from Sigma Aldrich/Fluka Analytical (Zwijndrecht, the Netherlands) and Merck Group (Darmstadt, Germany). The deuterated internal standards were acquired from Toronto Research Chemicals (North York, Canada). Chemicals needed for LC-MS/MS analysis, formic acid (FA), methanol (MeOH) and acetonitrile (ACN) were purchased at Merck Group (Darmstadt, Germany) and Boom B.V. (Meppel, the Netherlands) respectively. Ammonium formate (NH4HCO2, ≥ 99% HPLC) needed for buffer solution, was retrieved from Aldrich/Fluka Analytical (Zwijndrecht, the Netherlands).

Pharmaceutical extraction and analysis

For the analytical determination of micropollutant concentrations in wastewater, an optimized method was developed in collaboration with RadboudUMC. Samples (2.0 mL) were taken at time-points 0, 4, 8, 24, 48 and 96 hours after the start of incubation. Wastewater samples were centrifuged and the supernatant was stored at -20 °C until chemical analysis. To recovery the analytes present in the wastewater, solid phase extraction (SPE) was performed using Oasis HLB 3cc SPE cartridges (sorbent bed of 60 mg from Waters Corporation (Milford, USA)). First an acidic buffer solution with a of pH ~2.2 was prepared, by dissolving 10 mM NH4HCO2, and 3.25 mL FA in 250.0 mL Milli-Q water. Subsequently the SPE cartridges were conditioned with 2.0 mL of MeOH and flushed with 2.0 mL of acidic buffer solution prior to sample loading. SPE extraction was applied on 1.0 mL supernatant, spiked with 0.1 mL of deuterated internal standards (50 μg/L in methanol), diluted in 4.0 mL acidic buffer. Afterwards, 5.0 mL of MeOH was used to elute the retained OMPs. Vacuum was applied on each step. Next, the extracts were evaporated under a moderate stream of nitrogen using a sample concentrator set at 40 oC. Sample extracts were reconstituted in 0.1 % v/v FA (mobile

phase A). To extract metformin, a liquid-liquid extraction was performed (as in Yoshida and Akane 1999) (SI 3). 0.1 mL Sodium dodecyl sulfate (2.0 mM) was added to 0.5 mL of wastewater including 1.0 mL ACN. The tubes were placed in the freezer at -20 °C for 30 min and the upper organic layer was taken for analysis. SPE and LLE recovery data was calculated dividing the measured yield concentration by the measured original concentration, see Equation 1

$Recovery\ (R\%) = (Concentration\ (yield)/Concentration\ (original))*100$ (Equation S1)

LC-MS/MS analysis

For the analysis of pharmaceuticals, Liquid chromatography tandem mass spectrometry analysis (LC-MS/MS) was used. The LC system was coupled to a Xevo TQ-S micro quadrupole mass spectrometer (MS). Electro spray ionization (ESI) was used during the analysis of the pharmaceuticals. LC-MS/MS analysis was operated in positive ion mode to obtain two Multiple reaction monitoring (MRM) transitions per compound. Chromatographic separation was done on an Acquity UPLC BEH C18 (2.1 mm x 100 mm, 1.7 µm) reversed phase column. The mobile phases consisted of eluent (A); 0.1 % v/v FA in Milli-Q water and eluent (B); 100% ACN. Injection volume was set on 2.5 µL, and the gradient program used a flow of 0.5 mL/min. The gradient program was started at 100% (A) for only 0.2 min, changing to 100% (B) at 2.0 min. The 100% (B) was continued till 4.3 min and then switched back 100% (A) at 4.5 min. The overall gradient program was applied for 8 min. Prior LC-MS/MS analysis, calibration standards (n = 6, 0.5 – 100.0 µg/L) were freshly prepared and extracted in the same way as the wastewater samples. TargetLynx LC-MS/MS data acquisition software (Waters Corporation Milford, USA) was used for the linear curve fitting. Furthermore, 1/X weighting was used to minimise the mathematical error for the for the lowest calibration points (Almeida et al. 2002). This weighing was selected because it gave the lowest standard errors for all the points in the calibration curve compared to $1/X^2$ or not weighing. Quadratic curve fitting with the same 1/X weighting (y = ax2 + bx + c) was applied for the quantification of metformin. According to (Liu et al. 2019), the nonlinearity of metformin calibration curve is produced by an interplay between the deuterated internal standard and the original compound. Optimised LC-MS/MS parameters are provided in Figure S1, Table S1, and S2.

SI 2. Biotransformation rate constants

Acetaminophen was either not transformed (low concentration treatment in the summer experiment) or completely transformed within 24 hours. After 24 hours, the concentration increased again in some replicates, which might be the case due to acetaminophen contamination that occurred during the solid phase extraction method, making it difficult to obtain precise measurements at low concentrations. Therefore, only part of the curve was used, excluding these replicates/timepoints to calculate k_b values. Furthermore, in order to estimate k_b values for acetaminophen with an exponential decay models (according to Equation 1), the first timepoint was replaced by the theoretical concentration of the spiked treatment (i.e. background concentration + 3 or 30 nM). This was because the first measurement of acetaminophen (t = 0 h, < 20 min after addition) in the spiked activated sludge treatments were much lower than the concentration in the inactivated sludge treatment or even < LOD, likely due to fast uptake processes. One outlier was removed in order to calculate the k_b for metformin in the winter experiment: a value of > 300 nM metformin in the AS3 treatment at time = 8 h, which was over a hundred times higher than the theoretically added concentration (3 nM), matching the other two technical replicates. This value was also outside the interquartile range and this was likely caused by technical sampling error.

SI 3. Quantitative PCR protocol

All qPCR reactions were performed using PerfeCTA Quanta master mix (Quanta Bio, Beverly, MA) and 96-well optical PCR plates (Bio-Rad Laboratories Veenendaal, the Netherlands) with optical adhesive covers (Applied Biosystems, Foster City, CA). All reactions were performed on a C1000 Touch thermal cycler equipped with a CFX96 Touch™ Real-Time PCR Detection System (Bio-Rad Laboratories, Veenendaal, the Netherlands). The qPCR total volume was 25 µL containing 1 µL DNA sample of 1 to 10 ng/µL, 0.5 µL of each

primer solution of 20 µM, 12.5 µL of Quanta master mix and 10.5 µL of autoclaved MiliQ water. Negative controls were added to each run by replacing the template with sterile Milli-Q water. All qPCR data was analyzed using the Bio-Rad CFX Manager version 3.0 (Bio-Rad Laboratories, Veenendaal, the Netherlands). The qPCR protocol consisted of initial denaturation at 95°C 3 min followed by 39 cycles of denaturation, annealing, and extending (95°C 30 sec, 60°C 30 sec, and 72°C 30 sec, respectively), and two final steps at 65°C 5 sec and 95°C 50 sec.

Figure S1. Recovery of pharmaceuticals spiked in Milli-Q and synthetic sewage water

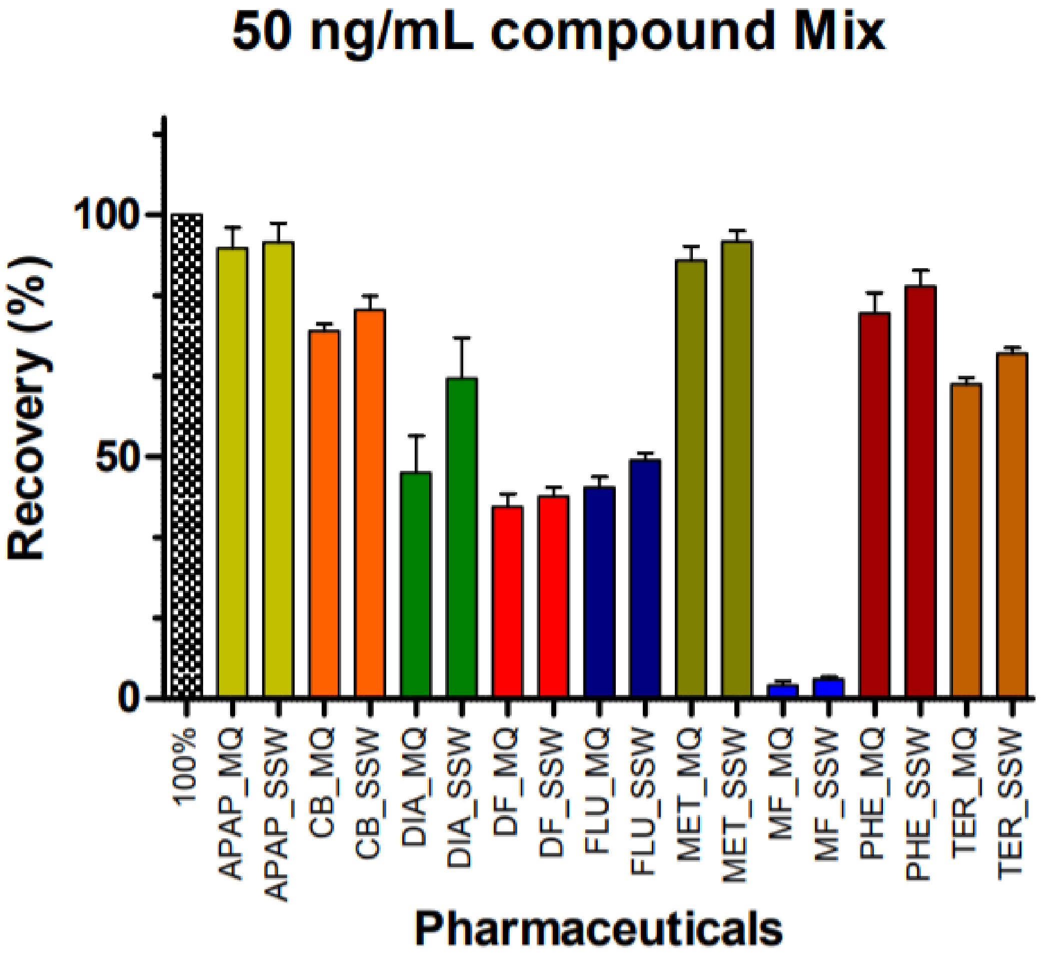

Figure S1. Recovery of pharmaceuticals spiked in Milli-Q and synthetic sewage water. A known concentration of 50 µg/L known concentrations, for the pharmaceuticals,

acetaminophen (APAP), carbamazepine (CB), diatrizoate (DIA), diclofenac (DF), Fluoxetine (FLU), metoprolol (MET), metformin (MF), phenazone (PHE) and terbutaline (TER).

Figure S2. Ammonium profiles during summer and winter experiments. Orange colour represents the experiment bottles with 30nM of each pharmaceutical added. Grey colour represents the bottles with doubled autoclaved biomass and 30nM of each pharmaceutical added. Blue colour in summer experiment represents the bottles with no pharmaceuticals added. Blue colour in winter experiment represents the bottles with 3 nM of each pharmaceutical added.

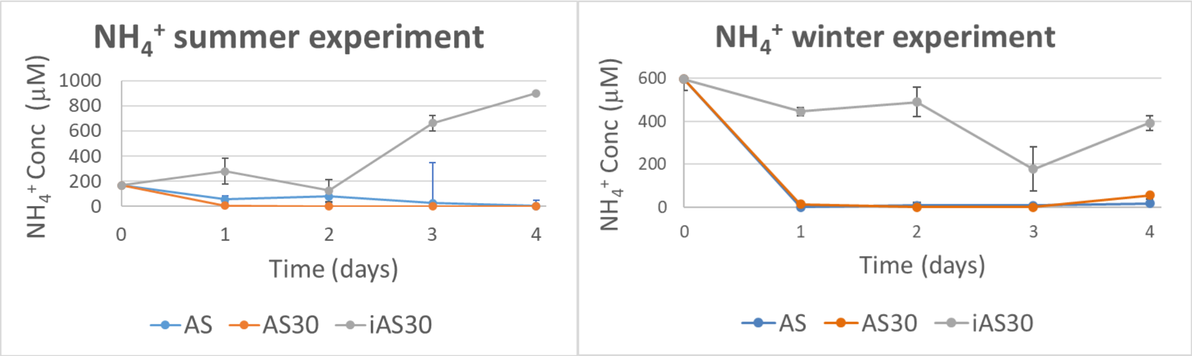

Figure S3. Nitrate profiles and production rates during summer and winter experiments. Nitrate production rates are calculated by fitting the dots to a line. In the summer experiment the rate is 67.448 µM*day^{-1} with a residual standard error = 53.919 on 13 degrees of freedom, p = 0.0097. In the winter experiment the rate is 90.265 µM*day^{-1} with a residual standard error = 25.4132 on 13 degrees of freedom, p = 2.63E-13.

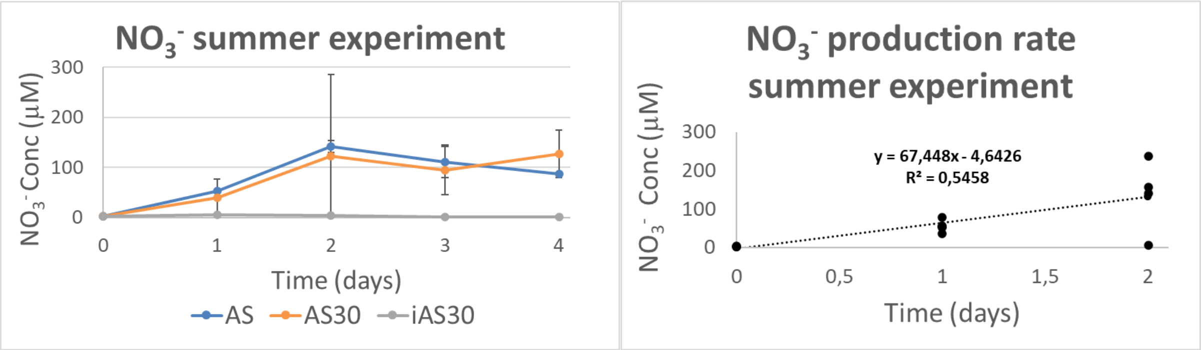

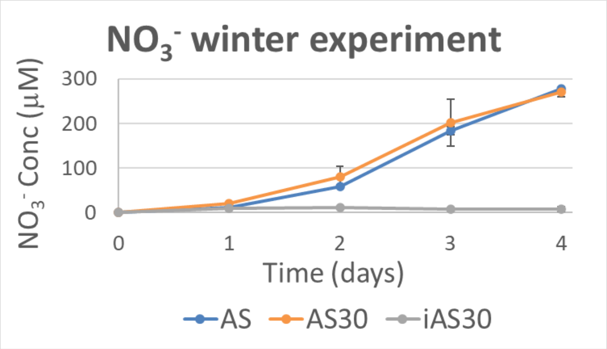
NO3- winter experiment
NO3- Conc (µM)
300
200
100
0
0
1
2
3
4
Time (days)
AS
AS30
iAS30

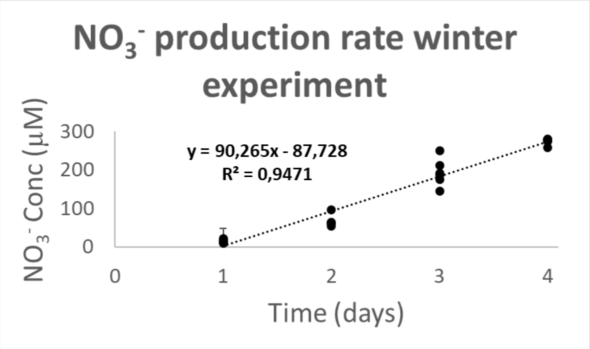
NO3- production rate winter experiment
y = 90,265x - 87,728
R² = 0,9471
NO3- Conc (µM)
300
200
100
0
0
1
2
3
4
Time (days)

Figure S4. Concentrations pharmaceuticals over time during the summer (a) and winter experiment (b). Graphs are shown for pharmaceuticals: acetaminophen, carbamazepine, diatrizoic acid, diclofenac, fluoxetine, metoprolol, metformin, terbutaline and phenazone. All measurement points are shown of three treatments: 1) activated sludge with no (summer; AS) or spiked with 3 nM of each pharmaceutical (winter; AS3); 2) activated sludge spiked with 30 nM of each pharmaceutical (AS30); and 3) inactivated sludge spiked with 30 nM of each pharmaceutical (iAS30). In some cases, no points are shown in case measurements were < LOD. Significant models are plotted in the graphs.

a) Concentration that was measured over time in the summer experiment

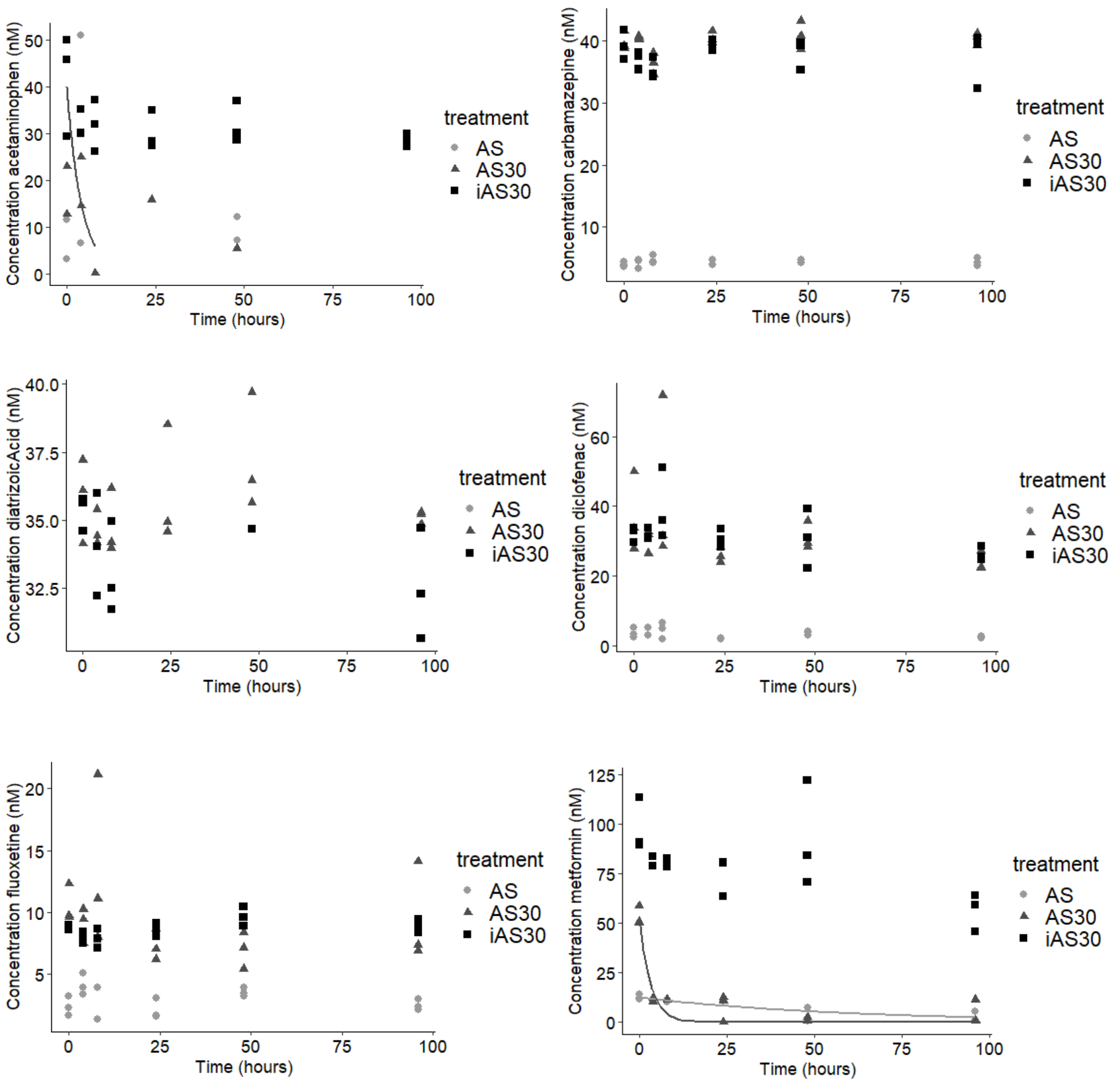

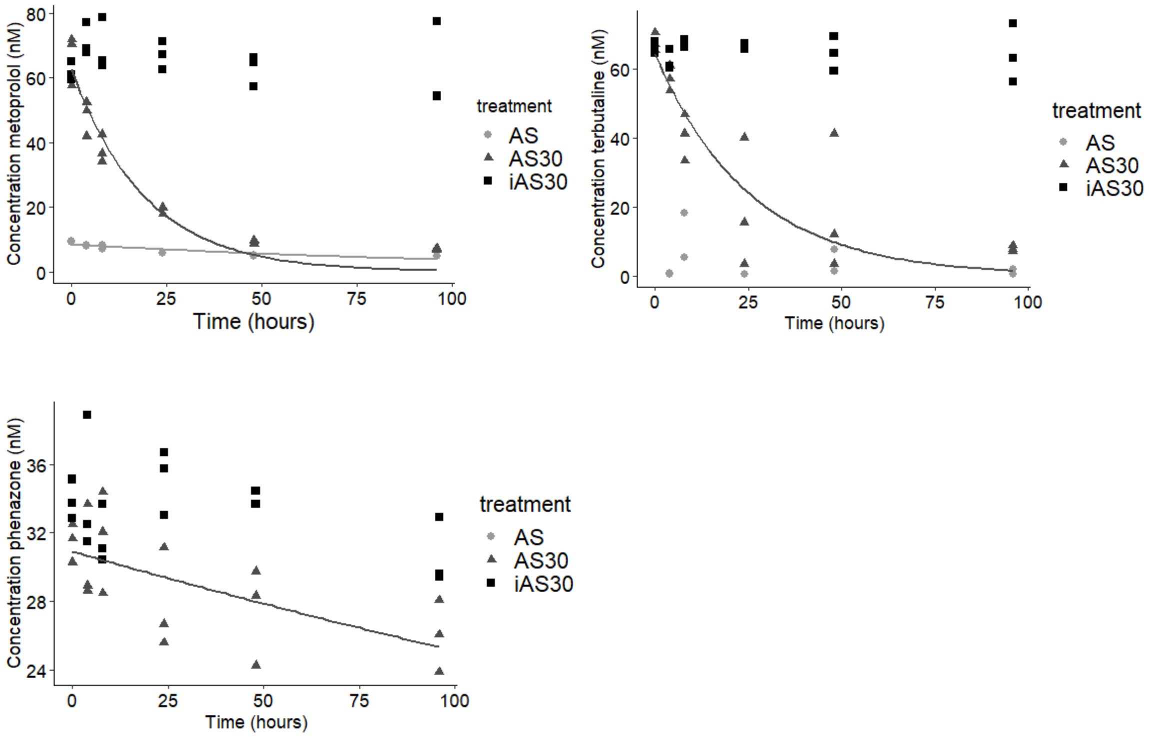

b) Concentration that was measured over time in the winter experiment

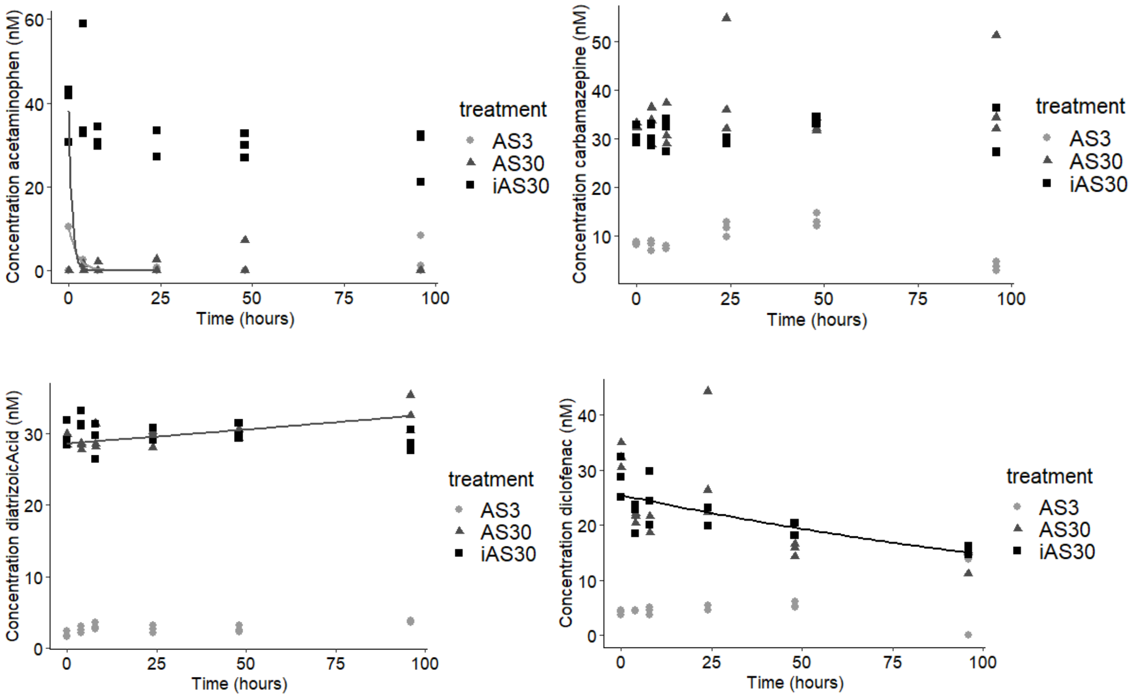

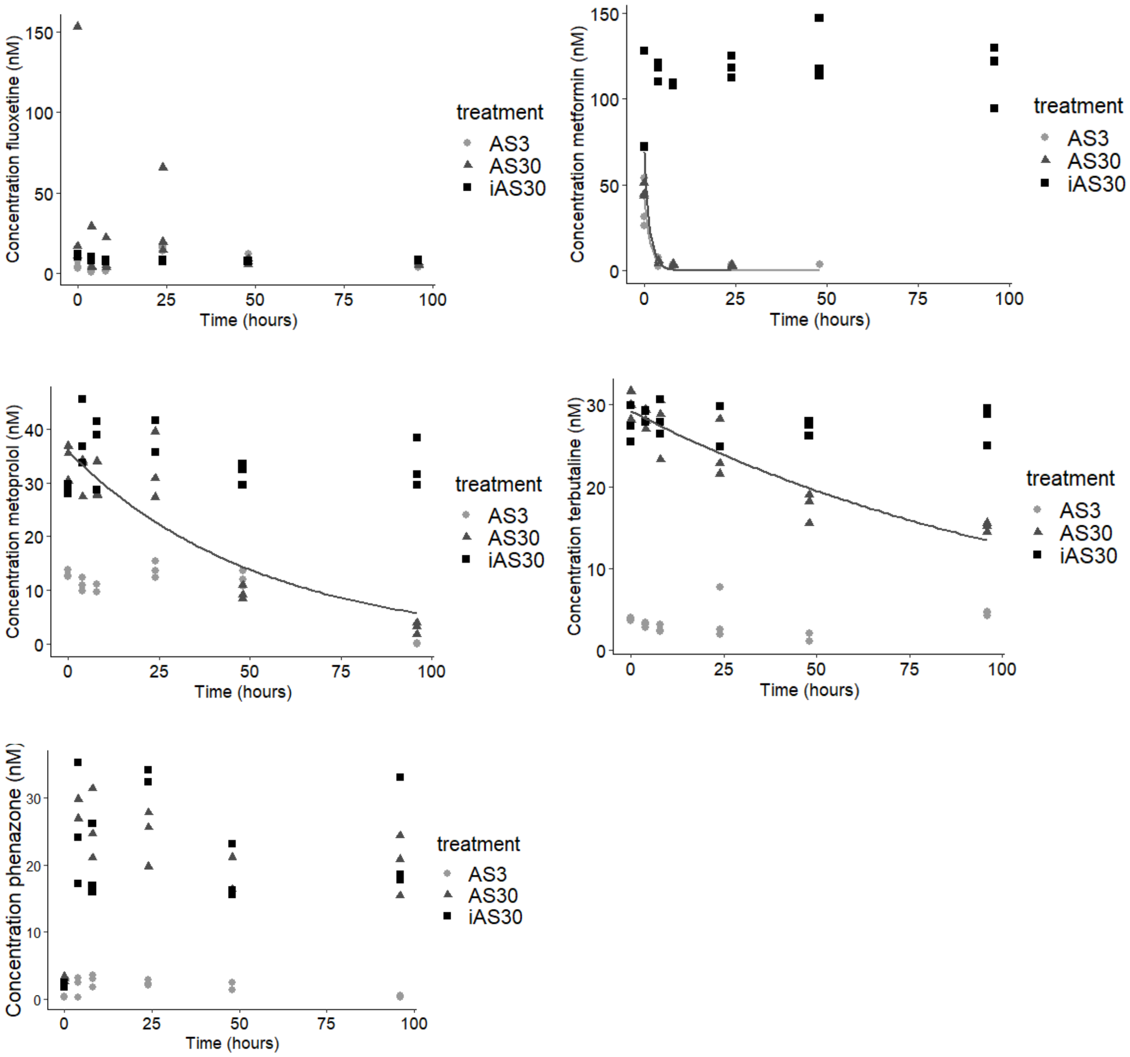
Concentration fluoxetine (nM)
Time (hours)
treatment
AS3
AS30
iAS30
Concentration metformin (nM)
Time (hours)
treatment
AS3
AS30
iAS30
Concentration metoprolol (nM)
Time (hours)
treatment
AS3
AS30
iAS30
Concentration terbutaline (nM)
Time (hours)
treatment
AS3
AS30
iAS30
Concentration phenazone (nM)
Time (hours)
treatment
AS3
AS30
iAS30

Figure S5. The pseudo-first order biotransformation rate constants k_b (log h^{-1}) plotted against initial pharmaceutical concentration (nM) at t = 0 of the experiment. If no error bar (SE) is visible in the figure, it was too small to show. Colors represent different pharmaceuticals, while circles indicate summer values and triangles indicate winter values.

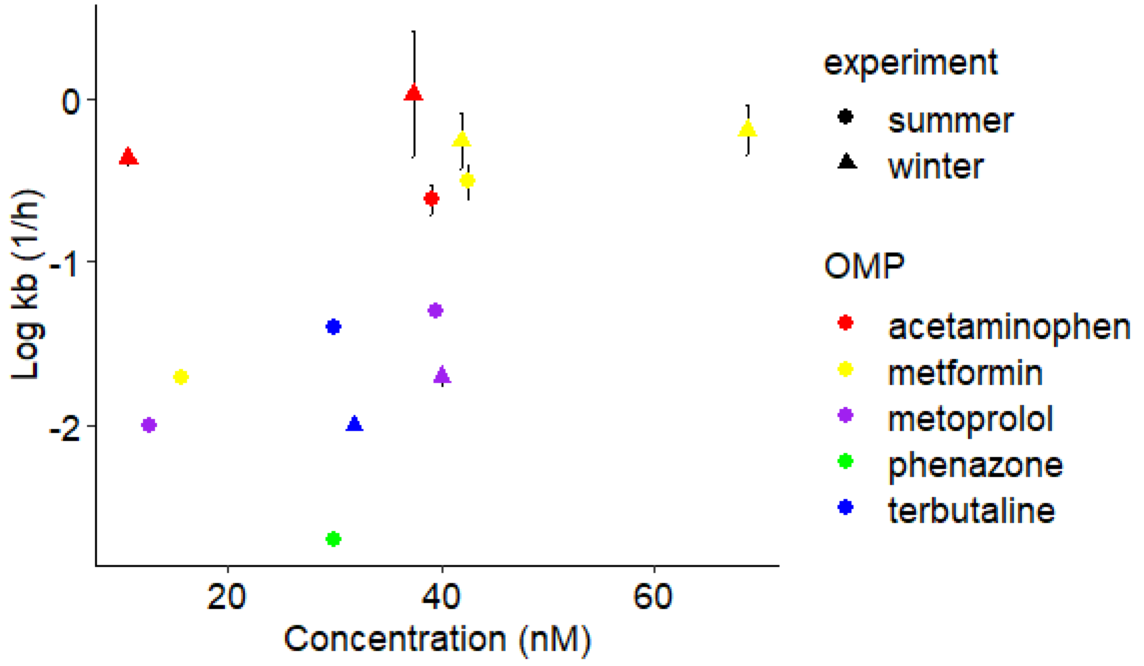

Figure S6. 16S rRNA gene relative abundance of putative acetaminophen degraders in summer and winter inocula

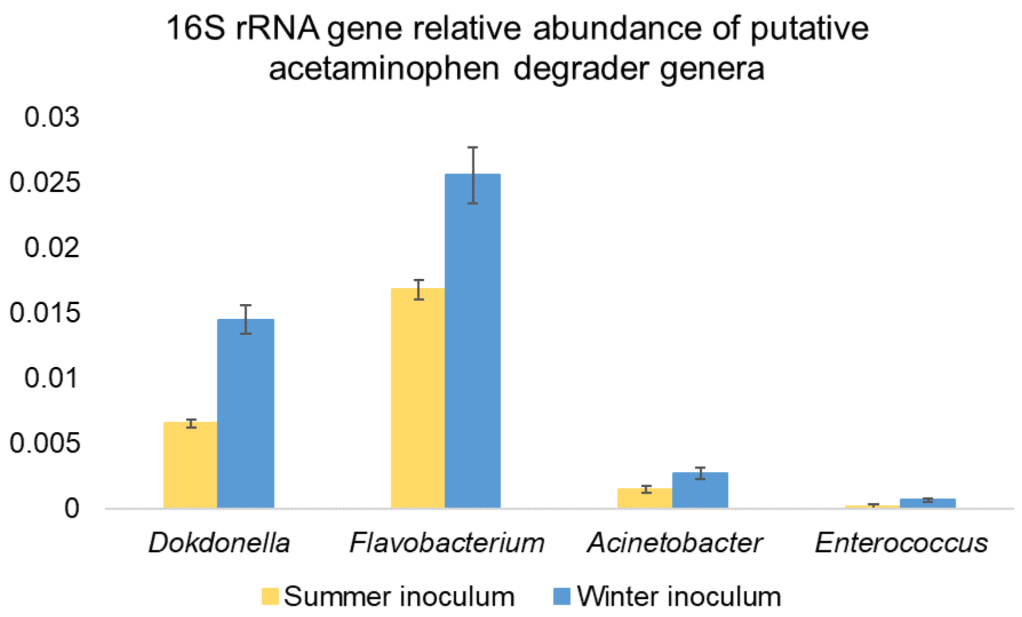

Figure S7. Relative cq value of bacterial *amo*A and 16S rRNA genes in summer and winter inocula and experiment bottles with different pharmaceutical concentrations.

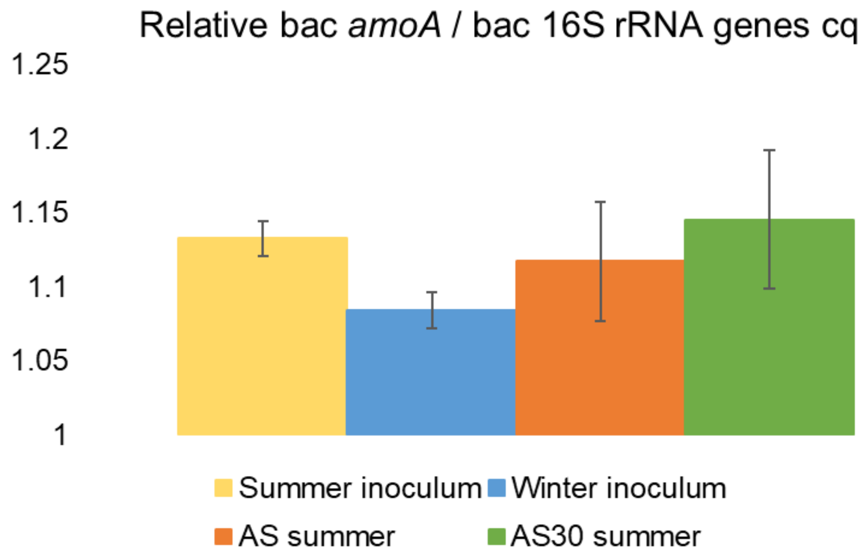

Table S1. Information on the pharmaceuticals selected in the experiment and the solid form that was used to obtain the dissolved spiking mixture. Log Kow is the octanol water partition coefficient

Pharmaceutical	Cas number	Class	Log K_{ow}	Solid form used for experiment	CAS number solid form	Molecular mass solid form (g/mol)	Supplier's name	Product number	Purity
Acetaminophen	103-90-2	Acid	0.46	Acetaminophen	103-90-2	151.2	Sigma Aldric	A7085	≥99%
Carbamazepine	298-46-4	Neutr al	2.45	Carbamazepine	298-46-4	236.3	Sigma Aldrich	C4024	100%
Diatrizoic acid	117-96-4	Acid	1.37	Diatrizoic acid	117-96-4	613.9	Sigma Aldrich	D9268	100%
Diclofenac	15307-86-5	Acid	1.85	Diclofenac sodium salt	15307-79-6	318.1	Sigma Aldrich	D6899	100%
Fluoxetine	54910-89-3	Base	1.51	Fluoxetine Hydrochloride	56296-78-7	345.8	Sigma Aldrich	PHR13 94	100%
Metformin	657-24-9	Base	-4.13	1,1 dimethylbiguanide hydrochloride	1115-70-4	165.6	Sigma Aldrich	D5035	≥ 97%
Metoprolol	37350-58-6	Base	1.88	Metoprolol Tartrate salt	56392-17-7	684.8	Sigma Aldrich	80337	100%
Phenazone	60-80-0	Base	0.38	Phenazone	60-80-0	188.2	Sigma Aldrich	A5882	100%
Terbutaline	23031-25-6	Base	0.9	Terbutaline Sulfate	23031-32-5	274.3	Sigma Aldrich	T00500 00	100%

Table S2. Optimization parameters LC-MS/MS and SPE extraction

**Compound**	**Retention** Time t_r (min)	**Transition** [M+H] (MRM)	**LOD**[A] (µg/L)	**Milli-Q Matrix** x̄ SPE Recovery (%) (±sd)	**Synthetic Sewage Water** x̄ SPE Recovery (%) (±sd)

				n = 3	n = 3
Acetaminophen	1.69	(151.968> 109.915) (151.968> 64.844)	0.24	93.2 (±7.3)	94.4 (±6.8)
Acetaminophen-d4	1.70	(156 > 69)	-	-	-
Carbamazepine	2.26	(236.968 > 178.876) (236.968 > 165.019)	<0.01	76.1 (±2.4)	80.2 (±5.8)
Carbamazepine-d10	2.27	(247 > 204)	-	-	-
Diatrizoate	1.63	(614.777 > 360.917) (614.777 > 233.018)	<0.01	46.8 (±13.2)	66.3 (±14.5)
Diatrizoate-d6	1.63	(621 > 367)	-	-	-
Diclofenac	2.66	(295.904 > 214.804) (295.904 > 150.951)	0.09	39.6 (±4.6)	41.9 (±3.0)
Diclofenac-d4	2.66	(299.9 > 155)	-	-	-
Fluoxetine	2.15	(310.224 > 148.035) (310.224 > 43.848)	0.03	43.7 (±3.8)	49.3 (±2.7)

Fluoxetine-d5	2.16	(315 > 153)	-		
Metoprolol	1.87	(268.266 > 116.031) (268.266 > 98.017)	<0.01	90.6 (±5.0)	94.5 (±3.8)
Metoprolol-d7	1.88	(275 > 123)	-	-	-
Metformin[B]	0.57	(129.968> 70.94) (129.968> 59.947)	0.11	1.3 (±0.7)	0.7 (±0.2)
Metformin-d6	0.56	(136 > 77)	-	-	-
Phenazone	1.94	(189.032 > 131) (189.032 > 103.878)	0.13	79.7 (±7.4)	85.4 (±4.9)
Phenazone-d3	1.95	(192 > 77)	-	-	-
Terbutaline	1.60	(226.224 > 170.019) (226.224 > 106.999)	<0.01	65.1 (±2.2)	85.4 (±4.9)
Terbutaline-d9	1.61	(235 > 153)	-	-	-

[A] **LOD** = Lowest calibration point detected * (3/ (*s/n*)), determined in Milli-Q 0.1% FA.

[B]: Quadratic Curve fitting used; $y = ax^2+bx+c$

Table S3. LLE extraction for metformin

LLE	Milli-Q Matrix	Synthetic Sewage Water

**Metformin**	**x̄ (R%) (±sd)**	**x̄ (R%) (±sd)**
50.0 µg/L (*n* = 3)	82.4 (±8.4)	64.9 (±4.7)

Table S4. Statistics of fitted biotransformation rate constants.

K_b is the pseudo-first order biotransformation rate constant and DF is degrees of freedom. AS is the activated sludge treatment without spiking (summer) or spiked with 3 nM of each pharmaceutical (winter), AS30 is the activated sludge treatment spiked with 30 nM of each pharmaceutical and iAS30 is the inactivated sludge treatment spiked with 30 nM of each pharmaceutical.

		Summer Exp.							Winter Exp.						
OMP	treatment	kb	SD	intercept	SD	p slope	residual standard error	DF	kb	SD	intercept	SD	p slope	residual standard error	DF
Acetaminophen	AS	ns	ns	ns	ns	ns	ns	ns	0.43	0.04	10.51	0.35	0.01	0.35	2
	AS30	0.24	0.09	39.94	7.1	0.23	7.23	1	1.07	0.39	37.98	0.80	0.11	0.80	2
	iAS30	ns	ns	ns	ns	ns	ns	ns	ns	ns	ns	ns	ns	ns	ns
Carbamazepine	AS	ns	ns	ns	ns	ns	ns	ns	ns	ns	ns	ns	ns	ns	ns
	AS30	ns	ns	ns	ns	ns	ns	ns	ns	ns	ns	ns	ns	ns	ns
	iAS30	ns	ns	ns	ns	ns	ns	ns	ns	ns	ns	ns	ns	ns	ns
Diatrizoate	AS	ns	ns	ns	ns	ns	ns	ns	ns	ns	ns	ns	ns	ns	ns
	AS30	ns	ns	ns	ns	ns	ns	ns	-0.001	0.0002	28.58	0.30	0.004	0.55	4
	iAS30	ns	ns	ns	ns	ns	ns	ns	ns	ns	ns	ns	ns	ns	ns
Diclofenac	AS	ns	ns	ns	ns	ns	ns	ns	ns	ns	ns	ns	ns	ns	ns
	AS30	ns	ns	ns	ns	ns	ns	ns	ns	ns	ns	ns	ns	ns	ns
	iAS30	ns	ns	ns	ns	ns	ns	ns	0.006	0.002	25.50	1.36	0.03	2.33	4
Fluoxetine	AS	ns	ns	ns	ns	ns	ns	ns	ns	ns	ns	ns	ns	ns	ns
	AS30	ns	ns	ns	ns	ns	ns	ns	ns	ns	ns	ns	ns	ns	ns
	iAS30	ns	ns	ns	ns	ns	ns	ns	ns	ns	ns	ns	ns	ns	ns
Metformin	AS	0.017	0.006	12.47	1.3	0.04	1.98	4	0.55	0.17	41.87	3.14	0.05	3.14	3
	AS30	0.31	0.1	52.77	6.15	0.04	6.09	4	0.64	0.15	68.88	3.09	0.05	3.09	2
	iAS30	ns	ns	ns	ns	ns	ns	ns	ns	ns	ns	ns	ns	ns	ns

Metoprolol	AS	0.008	0.003	8.48	0.59	0.03	0.96	4	ns	ns	ns	ns	ns	ns	ns
	AS30	0.05	0.01	62.64	4.34	0.01	5.01	4	0.02	0.01	35.80	3.75	0.03	5.78	4
	iAS30	ns	ns	ns	ns	ns	ns	ns	ns	ns	ns	ns	ns	ns	ns
Terbutaline	AS	ns	ns	ns	ns	ns	ns	ns	ns	ns	ns	ns	ns	ns	ns
	AS30	0.039	0.01	64.37	6.08	0.03	7.3	4	0.01	0.001	29.23	0.86	0.001	1.43	4
	iAS30	ns	ns	ns	ns	ns	ns	ns	ns	ns	ns	ns	ns	ns	ns
Phenazone	AS	ns	ns	ns	ns	ns	ns	ns	ns	ns	ns	ns	ns	ns	ns
	AS30	0.002	0.00005	30.91	0.65	0.02	1.15	4	ns	ns	ns	ns	ns	ns	ns
	iAS30	ns	ns	ns	ns	ns	ns	ns	ns	ns	ns	ns	ns	ns	ns

References

Almeida, A.M.d., Castel-Branco, M.M. and Falcao, A. (2002) Linear regression for calibration lines revisited: weighting schemes for bioanalytical methods. Journal of Chromatography B 774(2), 215-222.

Liu, Q., Jiang, F., Zhu, J., Zhong, G. and Huang, M. (2019) Development, Validation, and Application of a New Method To Correct the Nonlinearity Problem in LC-MS/MS Quantification Using Stable Isotope-Labeled Internal Standards. Anal Chem 91(15), 9616-9622.
